# Nonparametric identification of regulatory interactions from spatial and temporal gene expression data

**DOI:** 10.1186/1471-2105-11-413

**Published:** 2010-08-04

**Authors:** Anil Aswani, Soile VE Keränen, James Brown, Charless C Fowlkes, David W Knowles, Mark D Biggin, Peter Bickel, Claire J Tomlin

**Affiliations:** 1 Electrical Engineering and Computer Sciences, University of California, Berkeley, CA, USA; 2 Genomics and Life Sciences Division, Lawrence Berkeley National Laboratory, Berkeley, CA, USA; 3 Statistics, University of California, Berkeley, CA, USA; 4 Computer Science, University of California, Irvine, CA, USA

## Abstract

**Background:**

The correlation between the expression levels of transcription factors and their target genes can be used to infer interactions within animal regulatory networks, but current methods are limited in their ability to make correct predictions.

**Results:**

Here we describe a novel approach which uses nonparametric statistics to generate ordinary differential equation (ODE) models from expression data. Compared to other dynamical methods, our approach requires minimal information about the mathematical structure of the ODE; it does not use qualitative descriptions of interactions within the network; and it employs new statistics to protect against over-fitting. It generates spatio-temporal maps of factor activity, highlighting the times and spatial locations at which different regulators might affect target gene expression levels. We identify an ODE model for *eve *mRNA pattern formation in the *Drosophila melanogaster *blastoderm and show that this reproduces the experimental patterns well. Compared to a non-dynamic, spatial-correlation model, our ODE gives 59% better agreement to the experimentally measured pattern. Our model suggests that protein factors frequently have the potential to behave as both an activator and inhibitor for the same *cis*-regulatory module depending on the factors' concentration, and implies different modes of activation and repression.

**Conclusions:**

Our method provides an objective quantification of the regulatory potential of transcription factors in a network, is suitable for both low- and moderate-dimensional gene expression datasets, and includes improvements over existing dynamic and static models.

## Background

Inferring transcriptional regulatory networks in animals is challenging. For example, the large number of genes, the spatial and temporal complexity of expression patterns, and the presence of many redundant and indirect interactions all make it difficult to learn the network. In the long term, it will be necessary to use multiple data sets--including gene expression, genome wide *in vivo *DNA binding, and network perturbation data--to accurately represent all interactions. Combining multiple data classes in this way, however, is an open and challenging problem.

An alternative, intermediate approach is to use only gene expression data to infer regulatory networks. Here the relationships between the expression levels of one or more transcription factors and those of many putative target genes are used to predict which genes are the most likely targets of each factor. While much work has been done in this area, it is critical to understand the maximum amount of information that can be obtained about the network using this strategy.

Typical approaches for inferring regulatory networks have been to assume a model formulation and then fit the data to this formulation [1,2]. Many models have been proposed, including coexpression networks [3-5], information-theoretic representations [6-8], regression onto dynamical systems [9-14], Bayesian networks [15-17], and other graphical models [18,19], each of which has advantages and disadvantages. The primary differences between these models lie in the trade-off between statistical and interpretational issues. Techniques like Bayesian networks, graphical models, and information-theoretic models have protections against over-fitting (i.e., fitting models with many parameters to a small amount of experimental data); however, these techniques do not provide dynamical models which can generate new biological insights. On the other hand, techniques such as nonlinear regression and regression onto dynamical systems provide more biologically interpretable models, but sometimes suffer from inaccurate assumptions or over-fitting of the model to the data.

There is disagreement on the necessity of dynamical [9,8,15-19,14] as opposed to static [3,6,7,5,20-23] models. We feel that dynamical models are more philosophically pleasing because regulatory networks contain temporal characteristics: For example, a protein binds to DNA and initiates transcription, which eventually leads to transport of the mature mRNA to the cytoplasm. Yet the argument is often made that static models provide a quasi-steady-state interpretation of the network that may provide a sufficient approximation. Rigorous comparison of the two approaches, however, is lacking.

Dynamical modeling of animal regulatory networks has a long history [24,9,11,28]. It is a powerful approach in which researchers hypothesize a set of nonlinear, differential equations to describe the network, but it generally requires significant prior knowledge about the network. If there is insufficient biological knowledge about the network, then the structure of the equations can be incorrectly chosen. And if the model is not carefully chosen, it will have a large number of parameters, possibly leading to weak biological effects being erroneously identified as strong effects. Furthermore, it is sometimes shown that a wide range of different parameter values can reproduce the biological behavior of the network, which could be taken as evidence for either network robustness or over-fitting [26].

The purpose of this paper is to describe a novel approach for inferring regulatory networks from expression data, and it provides a new way to trade off statistical issues and model interpretability. We generate a quasi-genetic, formal model of regulatory networks using nonparametric ordinary differential equations (ODEs) which are fit using the nonparametric exterior derivative estimator (NEDE) [29,30]. For these reasons, we call our method and the resulting model the NODE (an amalgamation of NEDE and ODE) model. Our NODE model is similar to qualitative piece-wise linear network modeling and identification [13,12,14], and we extend these models by using identification techniques that have improved statistical properties and protect against over-fitting. The NEDE estimator adds constraints to the identification problem by learning correlations between factors, and these constraints protect the model from over-fitting and erroneously identifying weak biological effects as strong effects. Though we focus the discussion in this paper to temporal-spatial expression patterns, our NODE method can easily be used with time-series micro-array datasets. It is also scalable to a network sized on the order of hundreds of species.

We focus our modeling effort on the formation of *eve *mRNA stripes during Stage 5 of *Drosophila melanogaster *embryogenesis. We apply our technique to this portion of the regulatory network, and compare the performance of our method to that of other more commonly used models. We show that there are significant differences in the regulatory predictions made by the NODE model and other commonly used models, including the fact that our technique predicts that factors frequently have both positive and negative effects on the same targets, depending on the concentration of the factor. We also show that the NODE model performs better than a static, spatial-correlation model.

## Results and Discussion

Our NODE model is a formalization of a quasi-genetic model that seeks to capture the total net effect of direct and indirect influence of each factor on a target gene, and it is generated by looking at the correlation between factor concentrations and the *change *in target mRNA concentration over time. This is done in small windows of neighboring cells on the embryo and at different time intervals during development. By looking at the change in target mRNA over time, we are able to generate a dynamic equation model that describes each factors' influence on each gene in space and time; tuning parameters for our method are selected in a data-driven manner using cross-validation (see Methods and Models for more details). In general, the model formally predicts repression in all cases where increases in the concentration of a factor leads to a decrease in the rate of change in target mRNA over time. Similarly, it formally predicts activation as all instances where increasing the concentration of a factor leads to an increase in the rate of change in target mRNA over time.

**Figure 1:**
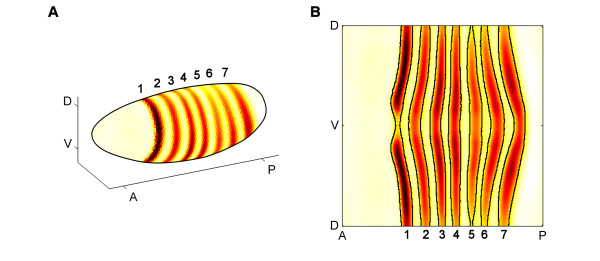
**Quantitative cellular resolution 3 D gene expression**. **A**. A three-dimensional plot of the *Drosophila *embryo showing the experimentally measured pattern of *eve *mRNA as it appears in late Stage 5. There are seven distinct expression stripes located along the anterior-posterior axis (AP) of the embryo, with the intensity of each stripe varying moderately along the dorsal-ventral axis (DV). **B**. A two-dimensional cylindrical projection of a Stage 5 *Drosophila *embryo provides an easier visualization of the details of the *eve *mRNA patterns, showing that expression of each stripe is similar on either side of the ventral mid line (V).

We applied our technique to experimental measurements, gathered by the Berkeley *Drosophila *Transcription Network Project (BDTNP), of spatial and temporal expression levels of transcription factor protein and mRNA in *Drosophila *embryos [31,20]. A NODE model was established that describes the formation of *eve *mRNA stripes during Stage 5 of development using data for five transcription factors known to be responsible for initiating much of the patterning of *eve*: Krüppel (KR), Giant (GT), Knirps (KNI), Hunchback (HB) and Bicoid (BCD) [21-23]. For each factor and for *eve *mRNA, there are 36,468 data points that represent 6,078 cells at 6 time points. Our technique was able to compute the model in approximately 20 hours on a desktop computer. The seven distinct *eve *mRNA stripes in the measured data can be seen in Figure 1, where both a three-dimensional view and a two-dimensional, cylindrical projection of the embryo are shown.

### Model fit

We assess the fit of our NODE model to the experimental data both qualitatively and quantitatively. Because we have an ODE model that describes the formation of the *eve *mRNA stripes, we can run a simulation of the model using only the experimentally measured *eve *concentration at the first time point of Stage 5 as the initial condition of the ODE. Only transcription factor protein and *eve *mRNA data from the first two time points was used to derive the NODE model for predicted regulatory interactions. By using this model along with the transcription factor protein expression data from all time points, we can then simulate the *eve *mRNA pattern for all six time points and then compare this to the experimentally measured *eve *pattern.

**Figure 2:**
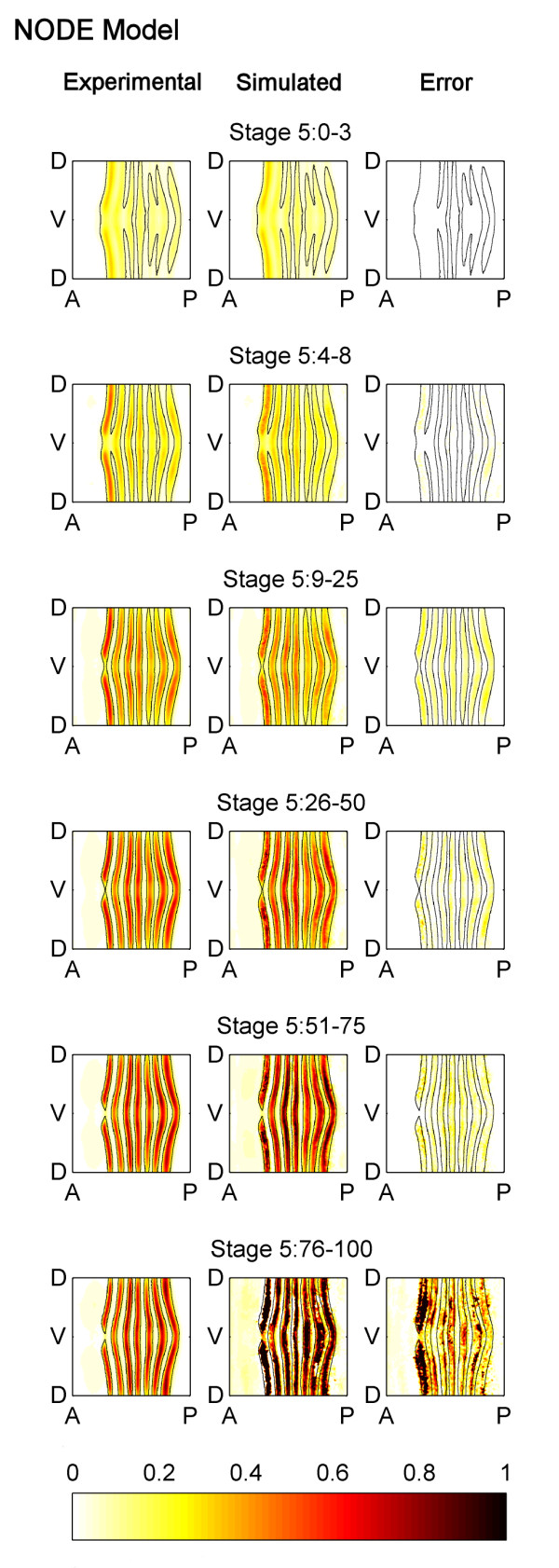
**Comparison of the experimentally measured and the NODE model simulated patterns of *eve *mRNA**. Cylindrical projections of the measured pattern of *eve *mRNA concentrations (left column), the NODE model simulated pattern of *eve *mRNA (center column), and the simulation error (right column) at six successive time points during blastoderm Stage 5 (rows). The *eve *mRNA concentration values have been normalized to range from 0 to 1 and the simulation error shown is the absolute value of the difference between experimental and simulated *eve *concentration in the embryo. The NODE model was generated using only data from Stage 5:0-3 and Stage 5:4-8, and the data from Stage 5:0-3 was used as the initial condition for simulation. It is able to predict the expression pattern well except for Stage 5:76-100.

Qualitatively speaking, the *eve *mRNA pattern generated by our NODE model simulation matches the temporal behavior of the experimental pattern quite well. The experimental and simulated *eve *patterns are compared in Figure 2. The black lines on each of the maps in Figure 2 show the boundaries of the experimental measurements of the *eve *mRNA stripes, and how they change location during Stage 5. Looking first at just the experimentally observed *eve *mRNA pattern shown in Figure 2, we can see that the stripe regions narrow, and *eve *concentration in the stripes becomes stronger. The stripes also shift anteriorly. The simulation of our NODE model matches this experimental behavior, and captures the changing boundaries of the *eve *stripes particularly well.

**Figure 3:**
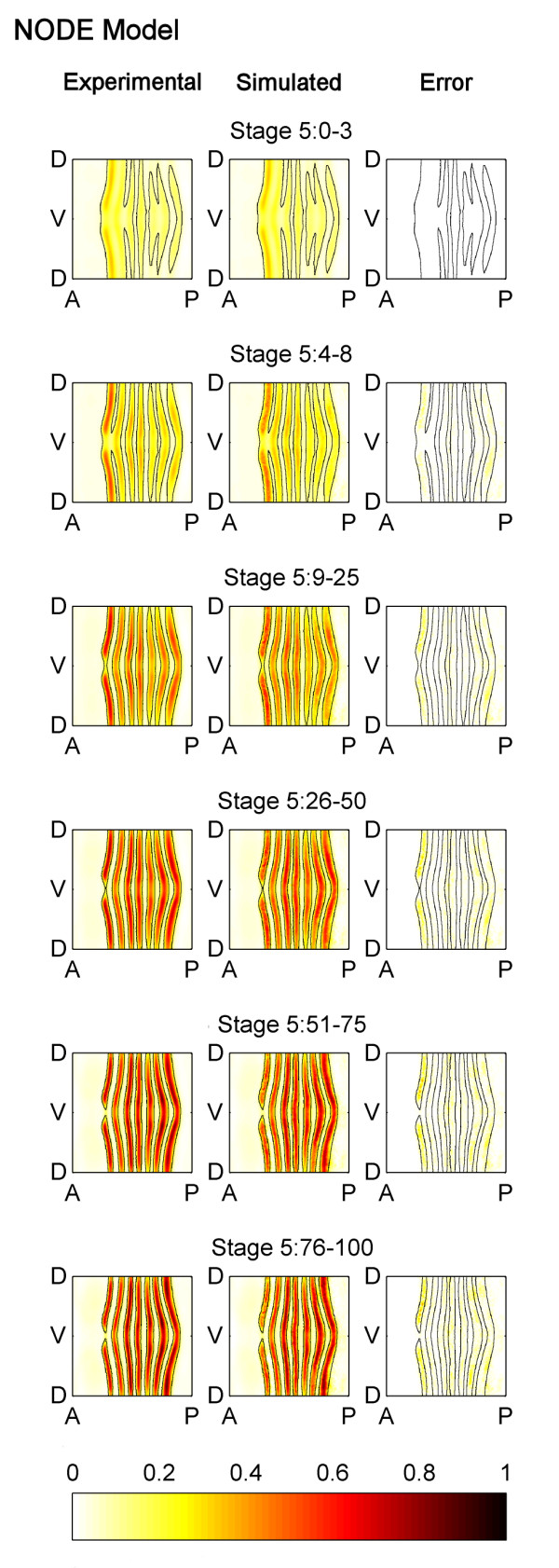
**Comparison of the experimentally measured and the NODE model (generated using *eve *mRNA expression from all time points) simulated patterns of *eve *mRNA**. A NODE model was generated using data from all time points in Stage 5, and it was used to predict the expression pattern. The simulation of this model shows better agreement with the experimentally observed pattern, than the NODE model shown in Figure 2 (which only uses two time points to generate the model). The figure is labelled using the same conventions as Figure 2 except that the simulation and error are for the NODE model which uses all time points.

To quantify the accuracy of the model, the simulation error is also shown in Figure 2. The NODE model is able to accurately predict the *eve *pattern at Stages 5:9-25, 5:26-50, and 5:51-75. Its predictions are less accurate for Stage 5:76-100 in some regions, especially in stripe 1, but this is not unexpected as it is known that at the end of Stage 5 a new set of transcription factors begin to regulate *eve *expression [32]. This could not have been learned using only data taken from early Stage 5 as we have done here. Indeed, if *eve *mRNA expression data from all time points is used to learn the NODE model, better agreement is seen (Figure 3).

### Factor activity plots

**Figure 4:**
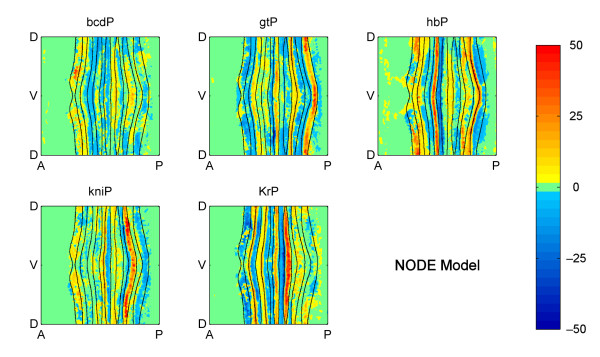
**Embryo wide factor activity at Stage 5:9-25 predicted by the NODE model**. Cylindrical projections of the correlation between each factor and the ***change ***in target expression over time. The intensity of the factor activity values is the product of the coefficients of the model in Equation 4 and the average, local factor concentration. The mathematical definition of factor activity is given in Methods and Models.

The model generated by our technique can be visualized as spatio-temporal maps of *factor activities*. An example of a spatial map for our NODE model for Stage 5:9-25 is shown in Figure 4, which shows how the five factors (directly or indirectly) affect *eve *mRNA pattern formation. Blue values correspond to predicted repression (i.e., an anticorrelation between factor expression and the rate of change of target expression) and yellow/red values correspond to predicted activation (i.e., a positive correlation between factor and the change in target).

Such factor activity plots show the intensity and variation of predicted effects of factors at different locations on the embryo and at different time points. Our model is a formal, quasi-genetic ODE model. It is not a mechanistic model, because it cannot capture the various mechanisms involved in the regulation of *eve *mRNA. This, however, is a strength because of the flexibility gained by not having to make *a priori *assumptions on the regulatory mechanisms. This comes at the cost of not being able to identify which interactions are direct or indirect.

### Comparison to spatial-correlation model

To aid understanding of our NODE model and help establish its utility, we compared it to a spatial-correlation model. Such models have also been used for identifying regulatory interactions from quantitative expression data [21-23,20,33], and are based on the descriptions of the relationship between transcription factor and target gene expression that have been most widely used by developmental biologists. These models are not dynamic and look at the correlation, at fixed time points, between factor concentrations and target mRNA concentrations. To make the result comparable to our NODE model, we consider a new variant of spatial-correlation models which looks separately at the correlation between factor levels and target mRNA levels in different, small regions of the embryo and at different stages of development.

**Figure 5:**
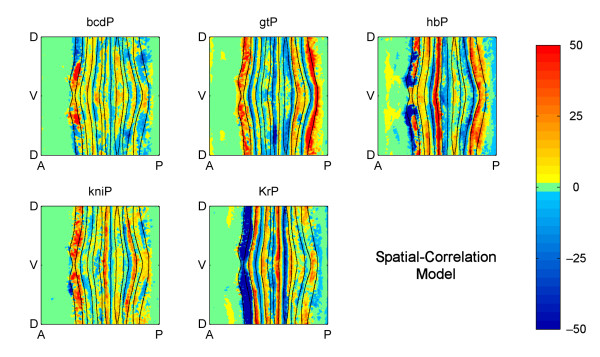
**Embryo wide factor activity at Stage 5:9-25 predicted by the spatial-correlation model**. Cylindrical projections of the correlations between each factor and the target expression. The intensity of the factor activity values is the product of the coefficients of the model in Equation 5 and the average, local factor concentration. The mathematical definition of factor activity is given in Methods and Models.

We first compared the embryo-wide spatial maps of factor activity in Figure 4 to that predicted by the spatial-correlation model (Figure 5). Viewed in this way, the two models show many similarities, which is encouraging because many experimentally validated regulatory interactions have been implicitly interpreted using a spatial-correlation model, and this agreement provides mutual support both for our model and the previously determined interactions.

**Figure 6:**
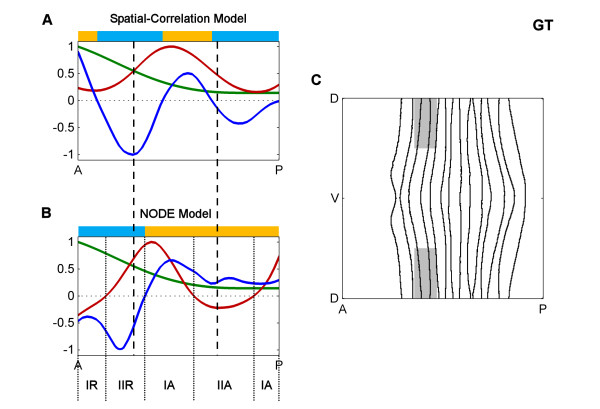
**Comparison of spatial-correlation and NODE models for GT at Stage 5:9-25**. **A**. The spatial correlation model along part of the anterior-posterior (AP) axis. Plotted are the concentrations of GT protein (green line) and *eve *mRNA (red line) as well as the factor activity of GT in the "spatial-correlation" model (dark blue line), calculated via a joint correlation of all factors with *eve *mRNA. The vertical dashed lines indicate the boundaries of *eve *stripe 2. The colored bars above indicate where the factor activity is positive (yellow) or negative (light blue). **B**. The NODE model along part of the AP axis. Plotted are the concentrations of GT protein (green line) and the change in *eve *mRNA over time (red line) as well as the factor activity of GT in the NODE model (dark blue line), calculated via a joint correlation of all factors with the change in *eve *mRNA. The vertical dashed lines indicate the boundaries of *eve *stripe 2. The regions of the embryo where GT is a type I or II activator or a type I or II repressor are indicated (IA, IIA, IR or IIR), and they are indicated with dotted lines. The colored bars above indicate where the factor activity is positive (yellow) or negative (light blue). **C**. The portion of the embryo that is plotted in A and B is shown in gray. The ventral region is omitted because otherwise the spatial variation of *eve *concentration along the dorsal-ventral (DV) axis makes interpretation of one-dimensional plots difficult. The values in the one-dimensional plots of A and B were generated by averaging over the DV axis and is done for strictly for visualization purposes. This averaging is not used in our standard analyses or method.

**Figure 7:**
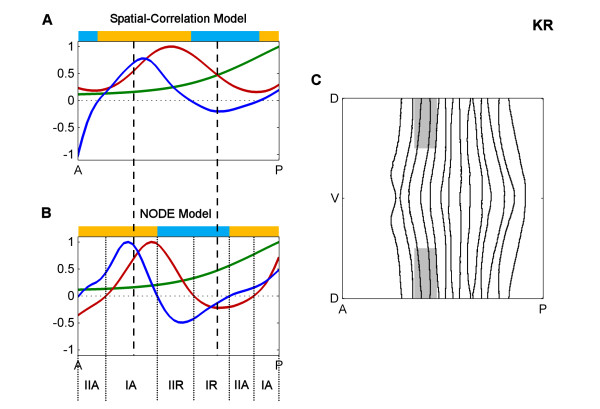
**Comparison of spatial correlation and NODE models for KR at Stage 5:9-25**. **A**. The spatial correlation model along part of the anterior-posterior (AP) axis. **B**. The NODE model along part of the AP axis. **C**. The portion of the embryo which is plotted in A and B. The figure is labeled using the same conventions as Figure 5 except that the protein expression and models are for KR protein.

Closer inspection, however, reveals significant differences in the precise locations of factor activity predicted by each method and, in some cases, differences in the direction of correlation at some stripes. To examine these in more detail, we next examined interactions during Stage 5:9-25 of two transcription factors, Giant (GT) and Krüppel (KR), with part of *eve *stripe 2 that other data suggest they repress (Figures 6 and 7) [23]. Figure 6A shows the concentrations of GT protein (green line) and *eve *mRNA (red line) along the anterior-posterior (AP) axis, showing the classic anti-correlation of GT protein with the anterior boundary of *eve *stripe 2. The factor activity predicted by the "spatial-correlation" model is shown as the plot of the GT correlation (dark blue line). In contrast, Figure 6B shows GT protein (green line) concentration; the change in *eve *mRNA concentration over time (red line); and the factor activity predicted by the NODE model for GT protein (dark blue line). While both models use the same protein expression data (green lines), the concentrations of *eve *mRNA and the temporal change in mRNA (red lines) show marked differences, as do the predicted factor activity profiles (dark blue lines). Similar differences are seen for KR (Figure 7).

**Figure 8:**
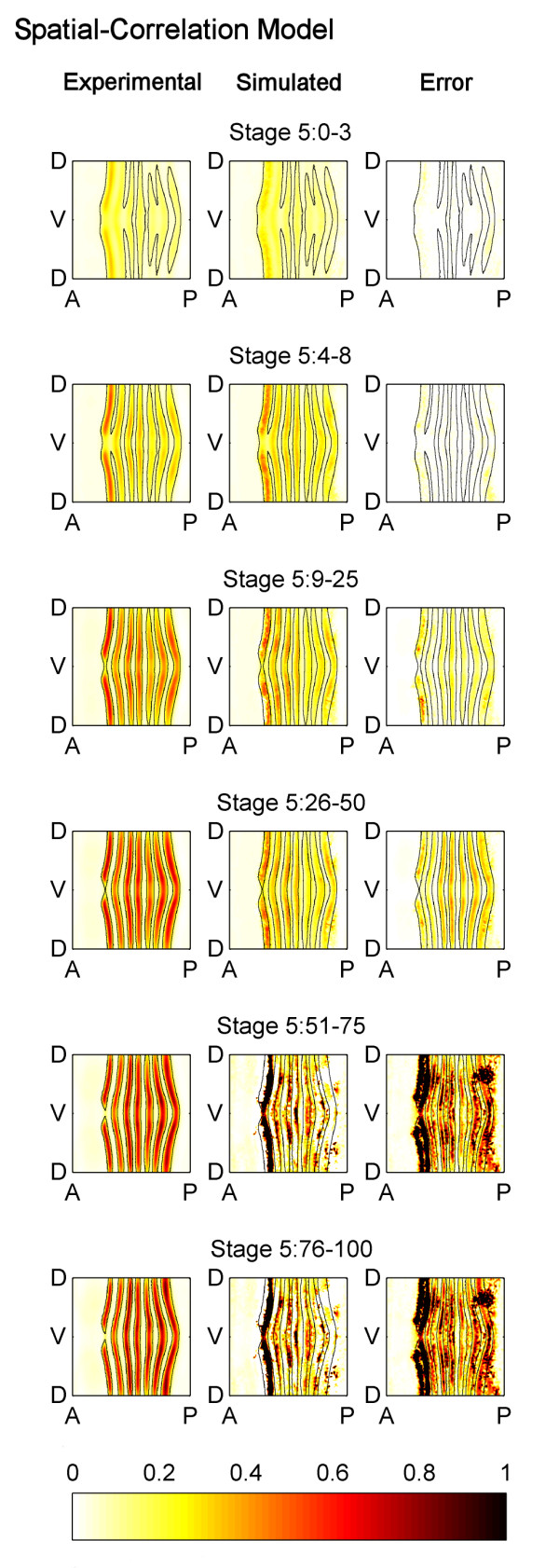
**Comparison of the experimentally measured and the spatial-correlation model simulated patterns of *eve *mRNA**. A spatial-correlation model was generated using only data from Stage 5:0-3 and Stage 5:4-8, and it was used to predict the expression pattern during later portions of Stage 5. The spatial-correlation model is unable to predict the expression pattern well, and is not as accurate as the NODE model which is shown in Figure 2. The figure is labelled using the same conventions as Figure 2 except that the simulation and error are for the spatial-correlation model.

These differences raise the question: Which model is more accurate and useful? To quantitatively compare the two models, we generated a spatial-correlation model which used *eve *mRNA expression data only from Stage 5:0-3 and Stage 5:4-8 and used it to predict the experimental *eve *pattern at later portions of Stage 5 (Figure 8). This spatial-correlation model much more poorly predicts the *eve *pattern at stages 5:9-25 and later. (Compare the error plots in Figure 2C with those in Figure 8C.) The NODE model predicts an *eve *pattern that has 59% less error over the last four time points than the pattern predicted by the spatial-correlation model. Thus, in a direct comparison of a static (spatial-correlation) model and a dynamical (NODE) model, the dynamic model is superior.

This result fits with the idea that the NODE model is intrinsically more biologically realistic than a spatial-correlation model. As stated earlier, biological networks are marked by temporal effects. For instance, a protein binds to DNA which initiates transcription. This is not an instantaneous process, and there is some delay between when a factor initiates transcription and when the target mRNA is expressed. The spatial-correlation model does not model this notion of temporal effects, whereas the NODE model does.

### Concentration-dependent effects

In many cases it is known that individual gene expression stripes can be controlled via a single *cis*-regulatory module (CRM) and current computational models generally assume that a given factor acts only as an activator or a repressor on a given CRM (e.g. [26,27,34-36]). However, both our NODE model and our variant of the spatial correlation model frequently predict concentration dependent effects whereby, on and around the same expression stripe, a factor has both repressing and activating effects (see the yellow and light blue bars above the plots in Figures 6 and 7 and more generally Figures 4 and 5). For example, consistent with previous molecular genetic evidence, KR is predicted as a repressor of posterior *eve *stripe 2, but is also implied by the model to be as an activator just anterior of this in cells where KR concentrations are lower (Figure 7). This and the many other similar cases could represent spurious correlations, perhaps due to other factors having dominant effects on targets in cells where the factor under study is expressed at lower levels. However, there are a number of cases where factors, including KR, have been shown to switch from activating to repressing the same target as their concentrations increase [37,38]. Thus, the predictions of both our NODE model and our variant of the spatial correlation model make it more obvious that gene regulation can involve multiple mechanisms of factor action that should be considered hence forth.

In some cases, the NODE model predicts factor activities that are closer to biological expectations than the spatial-correlation model. Figure 6 indicates that both models predict strong repression by GT in almost the same anterior portion of *eve *stripe 2 (regions where the blue lines are below 0). On the other hand, Figure 7 indicates that the spatial-correlation model predicts repression by KR mostly in the inter-stripe region between stripes 2 and 3, whereas the NODE model predicts repression by KR in the posterior half of stripe 2. Since it has been experimentally observed that the *eve *stripes narrow over time [31,20], and the NODE model more accurately indicates narrowing of the stripes, this provides further support for the idea that the NODE model performs better.

Another significant difference between the two models is that the NODE model can distinguish between multiple regions of the embryo where target mRNA either increases or decreases over time, whereas spatial-correlation models, by definition, cannot. This allows the NODE model to provide more subtle distinctions of factor activity.

We make the following formal definitions (see Methods and Models for the mathematical definitions):

• Type I Repression - At current factor concentrations, the target mRNA will decrease in concentration over time. An increase in factor concentration will lead to a faster rate of decrease in target mRNA amounts over time.

• Type II Repression - At current factor concentrations, the target mRNA will increase in concentration over time. An increase in factor concentration will lead to a slower rate of increase in target mRNA amounts over time.

• Type I Activation - At current factor concentrations, the target mRNA will increase in concentration over time. An increase in factor concentration will lead to a faster rate of increase in target mRNA amounts over time.

• Type II Activation - At current factor concentrations, the target mRNA will decrease in concentration over time. An increase in factor concentration will lead to a slower rate of decrease in target mRNA amounts over time.

**Figure 9:**
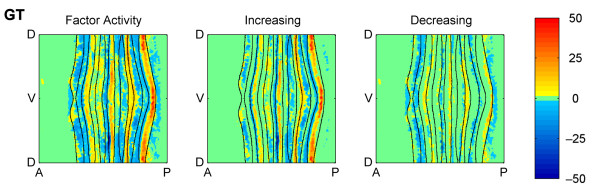
**Locations of type I and II activation and repression of *eve *by GT**. The factor activity of GT protein on *eve *as predicted by the NODE model is shown (left). The "Increasing" plot shows type I activation in yellow/red and type II repression in blue for cells where *eve *mRNA is increasing over time (center). The "Decreasing" plot shows type I repression in blue and type II activation in yellow/red for cells where *eve *mRNA is decreasing over time (right).

**Figure 10:**
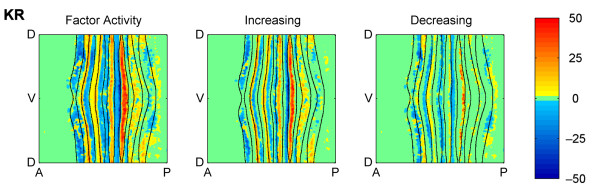
**Locations of type I and II activation and repression of *eve *by KR**. The figure is labelled using the same conventions as Figure 7 except that the models are for the factor activity of KR protein on *eve*.

With these definitions in hand we can readily see that, for example, while KR is a repressor within the posterior half of *eve *stripe 2, for most of this region it is a type II repressor, acting in cells where *eve *mRNA concentrations are increasing over time (Figure 9). Only in the very posterior margin of this stripe does the level of *eve *mRNA decrease. Similar distinctions between the two modes of activation and repression can be seen in embryo wide plots (Figures 9 and 10). The distinction between type I and II effects does not necessarily reflect different biochemical mechanisms between say, anti-activation and active repression, but equally they might. Certainly, the ability of the NODE model to make these distinctions provides a richer understanding of the relationship between factor and target expression than spatial-correlation models.

### Comparison to dynamical models

It is also instructive to compare our NODE model to existing dynamical models of spatial pattern regulation in *Drosophila *embryogenesis. There are dynamic models, some using nonlinear ODEs, that describe the developmental change in the expression of gap genes [26,27,35,36] and the *eve *stripes [34]. Some of these models only describe the network at the level of protein expression [26,27] whereas others include more detailed processes such as protein binding [34-36]. Like our model, these models can replicate experimentally measured gene expression patterns.

The models in [26,27] are similar to our work in some regards in that they concern the network at the expression level. However, they require significant biological knowledge in order to hypothesize the structural forms of their equations, which can be problematic because this limits their ability to provide new biological insights. For instance, an *a priori *biological assumption made by the models in [26,27] is that factors do not have concentration-dependent effects. A factor always either represses, activates, or does not affect the target gene. Biological experiments [37,38] and our models suggest that this is not always true.

The main disadvantage of the models in [34-36] is that they use *in vitro *data in fitting models for *in vivo *behavior. These models contain detailed predictions of the regulatory network such as levels of protein-DNA binding *in vivo*. This is problematic because the parameters of the models were calculated using only gene expression data and *in vitro *DNA binding data. No comparison was made between the models' inferences and actual measurements of *in vivo *DNA binding. Work by the BDTNP shows that there is no simple correlation between *in vitro *affinity and *in vivo *occupancy, even on highly bound functional targets [39]. This suggests that the models in [34-36] are unlikely to be accurate and that more quantitative data, such as ChIP-chip or ChIP-seq binding data, needs to be used to calculate the model parameters.

## Conclusions

We have described a novel approach for inferring interactions within animal regulatory networks. Our approach uses nonparametric statistics to generate ordinary differential equation (ODE) models from expression data, and it has certain statistical and mathematical advantages over existing approaches. It is able to generate spatio-temporal maps of factor activity, highlighting the times and spatial locations at which different regulators might affect target gene expression levels.

We identified an ODE model for *eve *mRNA pattern formation in the *Drosophila *blastoderm, and our model was able to reproduce the experimental patterns well. It gives 59% better agreement to the experimentally measured pattern, as compared to a spatial-correlation model. Our model suggests that protein factors frequently have the potential to behave as both an activator and inhibitor for the same *cis*-regulatory module depending on the factors' concentration, and implies different modes of activation and repression. This suggests further avenues of research.

## Methods

Here, we describe our NODE technique which uses time-series data to generate a dynamical model. We assume that the rate-limiting species (i.e. transcription factor protein concentrations) which drive the behavior of the network have been measured, and we do not consider actions on faster time-scales (e.g. the dynamics of factors binding to target genes). Also, we assume that concentrations are large enough for the rates of interaction to be deterministic.

Under these assumptions, the system can be reasonably described by an ODE:

(1) $$\frac{dx}{dt}=f(x),$$

where *x *is a vector whose elements are the concentrations of the rate-limiting species. Nonlinear regression techniques [2,1] start with a function with unknown coefficients, and then they regress the data onto this function. This is problematic because the relationships are highly nonlinear and one risks over-fitting the data by starting with a function with many unknown coefficients. In contrast, our NODE method does not make any assumptions on the functional form of *f*(*x*). We use nonparametric statistics to make local estimates of the ODE in Equation 1, and our tools can scale to networks with hundreds of species.

We focus our presentation on NODE models which describe the effect of five regulatory transcription factors on target *eve *mRNA, and we briefly comment on how this technique can be used with general, time-series data. The data set we use, code for our methods, and the models generated by our methods are publically available and can be downloaded from http://bdtnp.lbl.gov/Fly-Net/bioimaging.jsp?w=node.

### Experimental data

We apply our technique to experimental data that has been collected and processed by the BDTNP [31,20], where measurements of protein and mRNA concentrations are taken by analyzing images of many *Drosophila *embryos to create a virtual embryo. The virtual embryo consists of 6078 cells and is a computational, spatial decomposition which is determined by averaging the geometry and number of cells of different embryos [31,20]. The virtual embryo has measurements of the concentration (averaged over the different embryos at fixed points in time) of various protein factors and target mRNAs at the cellular level for six different time points during Stage 5 of the *Drosophila *embryo. We denote the vector of factor concentrations as *x*[*t*, *e*] and the vector of target gene concentrations as *y*[*t*, *e*], where *t *= 1, ..., 6 is the time of the measurement and *e *= 1, ..., 6078 is an index which uniquely identifies each cell in the virtual embryo. Notation like *x*_*bcd*_[*t*, *e*] denotes the [*bcd*] concentration in cell *e *at time *t*.

### Computational and statistical methods

The NODE technique is summarized in the following algorithm. Any tuning parameters are chosen in a data-driven manner using cross-validation [40,29,30].

Inputs: Factor concentrations *x*[*t*, *e*], target gene concentrations *y*[*t*, *e*]

Outputs: NODE model

1) Presmooth the factor concentrations *x*[*t*, *e*] and then compute time derivatives of the target gene concentrations *y*[*t*, *e*]

a) For each *e *= 1, ..., 6078

i) Do a least-squares fit of the polynomial $\overset{\wedge }{x}[t,e]=c_{0}+c_{1}t+...+c_{r}t^{r}$ (where *c*_0_,..., *c*_*r *_are coefficients and *r *is a tuning parameter) with the data points: *x*[*t*, *e*], for each *t *= 1, ..., 6

ii) Do a least-squares fit of the polynomial $\overset{\wedge }{y}[t,e]=k_{0}+k_{1}t+...+k_{r}t^{r}$ (where *k*_0_, ..., *k*_*r *_are coefficients and *r *is a tuning parameter) with the data points: *y*[*t*, *e*], for each *t *= 1, ..., 6

b) Presmoothed factor concentration data is given by $\overset{\wedge }{x}[t,e]$, and time derivative of target gene data is given by $d\overset{\wedge }{y}/dt[t,e]=k_{1}+k_{2}t\;...+k_{r}t^{r-1}$

2) Define matrix *Y *with rows given by $\left(d\overset{\wedge }{y}/dt[t,e]\right)$, for each *t *= 1, ..., 6 and *e *= 1, ..., 6078

3) Calculate the NODE model

a) For each *t *= 1, ..., 6 and *e *= 1, ..., 6078

i) Define matrix *X*_[*t*, *e*] _= [1 Ξ_[*t*, *e*]_], where first column is all one's and Ξ_[*t*, *e*] _is matrix with rows given by $\left(\overset{\wedge }{x}[u,v]-\overset{\wedge }{x}[t,e]\right)$, for each *u *= 1, ..., 6 and *v *= 1, ..., 6078

ii) Define weighting matrix *W*_[*t*, *e*] _to be diagonal matrix with entries along diagonal given by

(2) $$w[u,v]=\{\begin{array}{cc} 3\left(1-{\left(n[u,v]/h\right)}^{2}\right)/4, & \text{if }n[u,v]\leq h\\ 0 & \text{otherwise} \end{array}$$

for each *u *= 1, ..., 6 and *v *= 1, ..., 6078, where *n*[*u*, *v*] = ||*x*[*u*, *v*]-*x*[*t*, *e*]||_2 _is the Euclidean distance and *h *is a tunable parameter

iii) Define matrix *P*_[*t*, *e*] _by making its columns be the (*p *- *d*) principal components of Ξ_[*t*, *e*]_^*T *^*W*_[*t*, *e*]_Ξ_[*t*, *e*] _with smallest eigenvalues, where *p *is number of factors (*p *= 5 for the NODE model of target *eve *mRNA) and *d *is a tuning parameter

iv) Coefficients of NODE model, for *e*-th cell at *t*-th time point, are given by NEDE estimator

(3) $$\begin{array}{l} {\left[\begin{array}{cccc} b_{\left[t,e\right]} & a_{bcd,[t,e]} & ... & a_{Kr,[t,e]} \end{array}\right]}^{T}=\\ \;\;\arg\underset{\beta }{\min}{\left\|W_{\left[t,e\right]}{}^{1/2}\left(Y-X_{\left[t,e\right]}\beta \right)\right\|}_{2}{}^{2}+\lambda {\left\|P_{\left[t,e\right]}\beta \right\|}_{2}{}^{2} \end{array}$$

where

(4) $$\begin{array}{ll} \frac{d[eve]}{dt} & =a_{bcd,[t,e]}\left(\left[bcd]-{\overset{\wedge }{x}}_{bcd}[t,e\right]\right)\\ & +...\;a_{Kr,[t,e]}\left(\left[Kr]-{\hat{x}}_{Kr}[t,e\right]\right)+b_{\left[t,e\right]}. \end{array}$$

Step 1 involves presmoothing the experimental data and computing its time derivatives. We prefer to do this with local polynomial regression (LPR) [41] because it suffers from fewer transient effects than digital filters [42,41]. To simplify the presentation, Step 1.a describes polynomial regression (PR). LPR is a variant of PR which protects against over-smoothing the data, and it can be quickly computed by doing a weighted linear regression. More details on LPR can be found in [41].

This step is important because otherwise the NODE model will be statistically biased [43]. However, caution must be used when deciding to presmooth certain data sets in which the measurements are very noisy and taken at a sparse grid of points in time. In such cases, there is a risk of smoothing out biologically-relevant, temporal trends in the data because of the sparsity of the temporal grid.

Step 3 computes the NODE model, and we make use of the NEDE estimator: a new statistical tool that protects against over-fitting [30]. The computation in Step 3.a.ii determines a window of cells *v *at time u that have concentrations similar to cell *e *at time *t*. The size of this window is selected by the parameter *h*, and cells with highly (weakly) similar concentrations are weighted highly (weakly) in the estimation of the coefficients of the NODE model. Equation 2 uses the Epanechnikov kernel to do this weighting. Note that weights for cells with very different concentrations can be similar, because the Euclidean distances computed in Step 3.a.ii can be similar. This does not cause problems because the NEDE estimator has been proven to be statistically well-behaved in the presence of such weighting schemes [41,29,30].

Step 3.a.iv uses the NEDE estimator in Equation 3 to compute the coefficients of the NODE model. It protects against over-fitting by learning constraints which the data obeys (Step 3.a.iii), and then using these constraints to reduce the degrees of freedom in the regression. In general, the data points *x*_[*t*, *e*] _form a manifold, and the projection matrix *P*_[*t*, *e*] _in Equation 3 enforces that the regression coefficients lie close to the manifold. This methodology is motivated by differential geometry which says that the exterior derivative of a function on an embedded submanifold lies in the cotangent space [44,29,30]. The NEDE estimator can be calculated quickly on a computer because it is a convex optimization problem. Theoretical properties and a more detailed description of the NEDE estimator can be found in [29,30].

### NODE model interpretation

Instead of using a single ODE model to describe the regulatory network, the NODE model uses a group of ODE models consisting of the first order Taylor expansion (i.e., linearization) of the ODE given in Equation 1. Each equation of the NODE model describes how the behaviour of the regulatory network changes if concentrations of the factors in cell *e *at time *t *are changed. It requires fewer assumptions or prior knowledge about the system, because it does not require knowing the mathematical structure of the single ODE model in Equation 1. The disadvantage of this approach is that it is more difficult to interpret a series of models. The full NODE model for formation of target *eve *mRNA is given by Equation 4, and there is a different equation for each cell *e *at time *t*. Though the NEDE estimator protects against over-fitting, some might feel that the NODE model over-fits. The predictive ability of the NODE model, as discussed in Results and Discussion, gives evidence that it does not over-fit. In that test, we used our algorithm on the first two time points of data, and we assumed that the model for cell *e *at times *t *= 3, 4, 5 was the same as the model for cell *e *at time *t *= 2.

Equation 4 is difficult to interpret because the coefficients vary depending on cell *e *at time *t*, due to the fact that each equation is a linearization that is valid for when factor concentrations are close to *x*[*t*, *e*]. The model describes how a change in factor concentrations in the presence of all factors (the right hand side of Equation 4), affects the change in time of *eve *(the left hand side of Equation 4). If *d*[*eve*]/*dt *is positive (negative), then *eve *concentration will increase (decrease) by the next instant of time. For example, suppose the concentrations of all species are kept fixed at $\overset{\wedge }{x}[t,e]$ except for the concentration of GT which is slightly increased from ${\overset{\wedge }{x}}_{gt}[t,e]$ to $[gt]={\overset{\wedge }{x}}_{gt}[t,e]+\Delta gt$. In this situation, the change in time of *eve *concentration will be given by *d*[*eve*]/*dt *= *a*_*gt*,[*t*, *e*]_Δ*gt *+ *b*[*t*, *e*]. The increase of GT concentration by Δ*gt *leads to a change in the change in time of *eve *concentration by *a*_*gt*,[*t*, *e*]_Δ*gt *amount, and the sign of *a*_*gt*,[*t*, *e*] _describes whether this change is positive or negative.

Because this equation describes relationships in the presence of all factors, this can lead to seemingly contradictory results, such as when one species is a putative activator (e.g., BCD protein upregulates *eve *mRNA), but increasing the concentration of the activator in the presence of the other species can have a slight repressive effect because of interactions between the activator and the other factor species (i.e., the described concentration-dependent effects). Such a situation leads to an odd result: The coefficient of the "activator" will be negative.

Our NODE model is different from the spatial-correlation model [20,33-23]. We consider the following version of the spatial-correlation model:

(5) $$\begin{array}{ll} \left[eve\right] & =a_{bcd,[t,e]}\left(\left[bcd]-{\overset{\wedge }{x}}_{bcd}[t,e\right]\right)\\ & \;\;+...\;a_{Kr,[t,e]}\left(\left[Kr]-{\overset{\wedge }{x}}_{Kr}[t,e\right]\right)+b_{\left[t,e\right]}, \end{array}$$

and this model looks for the correlation of *eve *mRNA with protein factor concentrations. Whereas Equation 4 is a dynamical model, the model in Equation 5 is a static model, because it does not describe the temporal changes in *eve *concentration. A comparison of the fits between these models can be seen in Results and Discussion. Note that the coefficients in Equation 5 are computed with the algorithm for our NODE technique, with the change that *Y *is a vector of *eve *concentrations.

### Factor activity

Factor activity is a quantitative measure of the impact of a factor on the target gene expression, and it is a particular scaling of the coefficients (or correlations) of the model. It takes into account the concentration of the factors and the coefficients of Equation 4, which describe the amount of influence of the factors on the target expression. Without loss of generality, we give the equation for factor activity of GT on the expression of *eve *mRNA

(6) $$a_{gt,[t,e]}{\left(\frac{1}{n}{\left[\Xi_{\left[t,e\right]}{}^{T}W_{\left[t,e\right]}\Xi_{\left[t,e\right]}\right]}_{gt}\right)}^{1/2}.$$

The first term is the coefficient from Equation 4, and the second term in parenthesis is a measure of average GT concentration within cells whose factor concentrations are similar to cell *e *at time *t*. The second term in parenthesis in Equation 6 is a measure of average concentrations, because it is a measure of the mean difference from the baseline concentration of *x*[*t*, *e*]. To clarify the notation, suppose the *i*-th value of *x*[*t*, *e*] denotes: *x*_*gt*_[*t*, *e*], which is GT concentration. Then the term [Ξ_[*t*, *e*]_^*T *^*W*_[*t*, *e*]_Ξ_[*t*, *e*]_]_*gt *_denotes the *i*-th value along the diagonal of the matrix Ξ_[*t*, *e*]_^*T *^*W*_[*t*, *e*]_Ξ_[*t*, *e*]_.

For the NODE model, the factor activities can be subdivided into four categories of behavior. Without loss of generality, we provide mathematical definitions for four categories of GT activity on *eve *mRNA. At a given concentration *x*[*t*, *e*], if the GT coefficient from Equation 4 is negative (i.e., *a*_*gt*,[*t*, *e*] _< 0) and *eve *concentration is decreasing (i.e., *d*[*eve*]/*dt *< 0), then GT is formally a Type I repressor. A summary of the other mathematical definitions is given in Table 1.

**Table 1:** Mathematical definition of factor activity classification in the NODE model

	***sign*(*a***_***gt*,[*t*, *e*]**_**)**	*sign*(*d*[*eve*]/*dt*)
Type I Repression	-	-

Type II Repression	-	+

Type I Activation	+	+

Type II Activation	+	-

Without loss of generality, we consider the factor activity of GT on *eve*, as described by Equation 4. The classification is dependent on the mathematical sign of the coefficient of the model *a*_*gt*,[*t*, *e*] _and the mathematical sign of the change in *eve *mRNA *d*[*eve*]/*dt*, and it is different for each factor concentration *x*[*t*, *e*]. A positive (negative) sign is denoted with the "plus" ("minus") symbol "+" ("-").

### Window sizes

**Figure 11:**
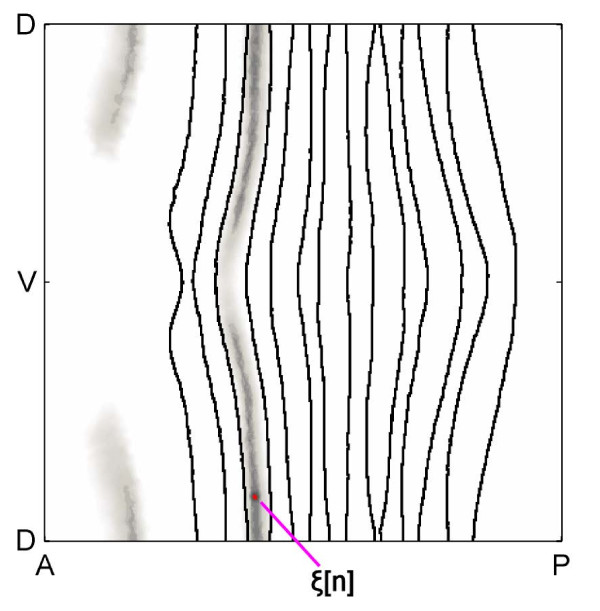
**Window of cells with similar concentrations**. The cell which represents *x*[*t*, *e*] is shown in red, and a purple line points towards this cell. The window of cells with similar factor concentrations is shown in gray, and cells farther away from the red-colored cell are less similar. Cells with more similar concentrations are shown by darker shades of gray, and cells not in the window are colored white. The black lines show the boundaries of the experimental *eve *pattern. The NODE method takes the amount of similarity of the cells into account when doing the regression procedure.

An example of a window is shown in Figure 11. The NODE method uses Equation 2 to take the similarity of the cells into account when doing the regression procedure. The size of the window is determined by the parameter *h *which is chosen using cross-validation, and it changes for each cell *e *at time *t*. As explained earlier, the statistical tools are well-behaved when weighting of cells within this window is computed with Euclidean distance.

Our method can automatically identify symmetries in the embryo patterns. The window contains cells on the other half of the embryo, because it can tell that the embryo has symmetry along the left-right axis. Similarly, it can divide the embryo into stripe-like regions which correlate to the positions of the *eve *stripes. This happens because our method looks for cells with factor concentrations similar to the red-colored cell, rather than just including cells spatially near the red-colored cell.

To check that window sizes selected in a data-driven manner were not too small and missing important features, we did a check in which we fixed the windows to surround cell *e *at time *t *with a circle of radius of three cells at time *t*. This size was chosen, because the *eve *stripes are about six cells wide at Stage 5. A circular window with this size would not miss important regulatory features of the network. The [Additional file 1] shows plots of factor activity as generated by our NODE method for both data-selected and fixed windows. A visual comparison of the factor activity plots generated by these two windows shows that the data-selected windows were able to identify the same features as the fixed, circular window.

### General time-series data

Our NODE technique can be applied to general time-series data. The NODE model is

(7) $$dx/dt=A_{\xi [n]}(x-\xi [n])+b_{\xi [n]},$$

where (a) *ξ*[*n*] for *n *= 1, ..., *N *is a user-selected set of linearization points of Equation 1, (b) *A*_ξ[*n*] _= *Df*(*ξ*[*n*]) and *b*_ξ[*n*] _= *f*(*ξ*[*n*]) are the coefficients of the model, and (c) *Df *is the gradient of *f*(*x*). The NODE technique is unchanged except *e *refers to different experiments (instead of different cells), and Equation 3 is applied column-wise to *Y *to give columns of the matrix of coefficients: [*b*_ξ[*n*]_^*T *^*A*_ξ[*n*]_*^T^*]*^T^*.

## Authors' contributions

AA carried out the modeling work, designed the computational methods, and drafted the manuscript. SVEK carried out the experimental work and assisted in analysis of the models. JB helped to refine the modeling. CCF and DWK processed the data to generate the virtual embryos. MDB helped conceive the experiments and draft the manuscript. PB and CJT aided in improving the computational methods. All authors read, revised, and approved the final manuscript.

## Supplementary Material

Additional file 1 **Supplementary material**. Full set of Factor Activity plots generated with both cross-validation-selected and fixed window sizes. Click here for file
