# Supplementary material for: Nonparametric identification of regulatory interactions from spatial and temporal gene expression data
Source: BMC Bioinformatics. 2010 Aug 4;11:413. doi: 10.1186/1471-2105-11-413 (PMC2933715; doi:10.1186/1471-2105-11-413)

Factor Activity of NODE Model with Auto-Selected Window Sizes

Stage 5:0-3

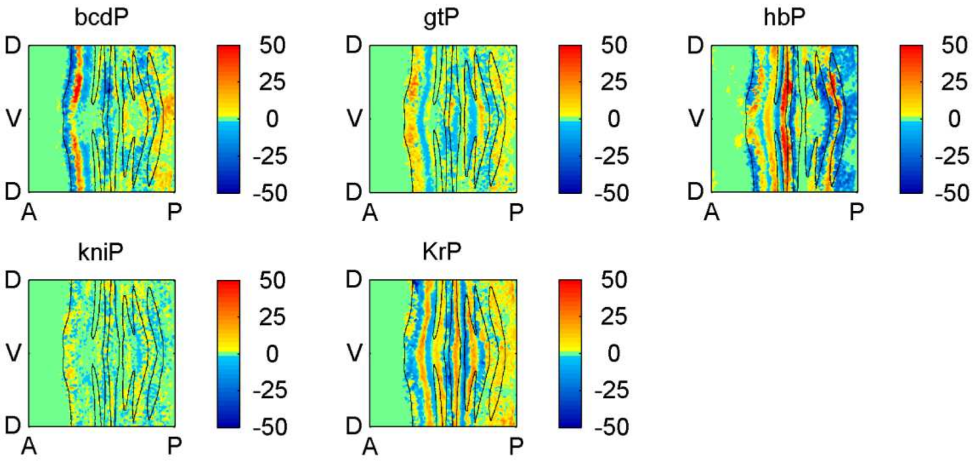

Stage 5:4-8

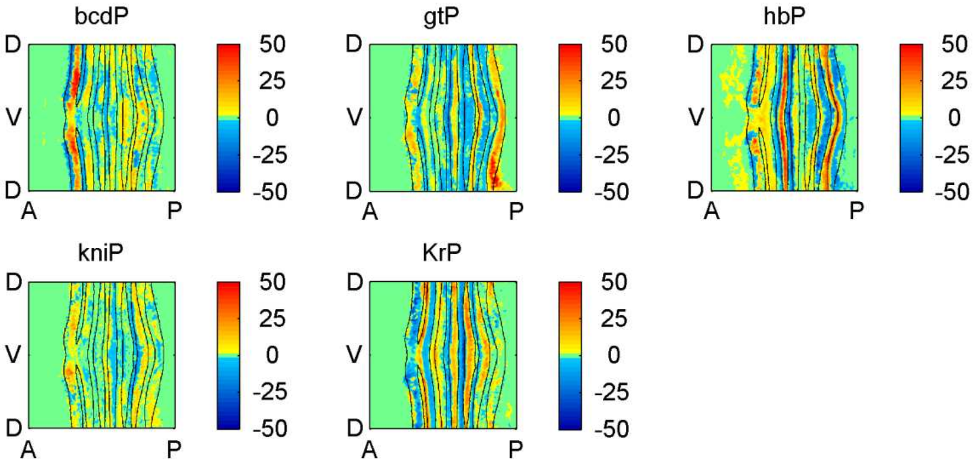

Stage 5:9-25

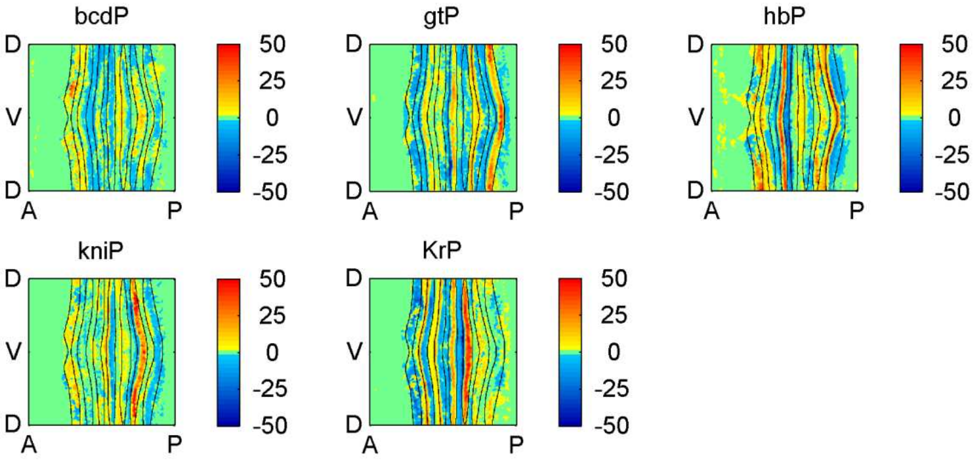

Stage 5:26-50

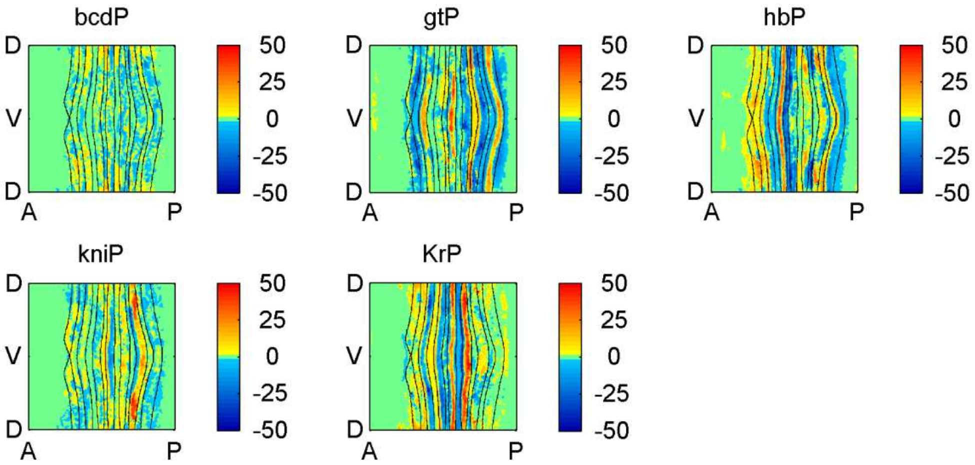

Stage 5:51-75

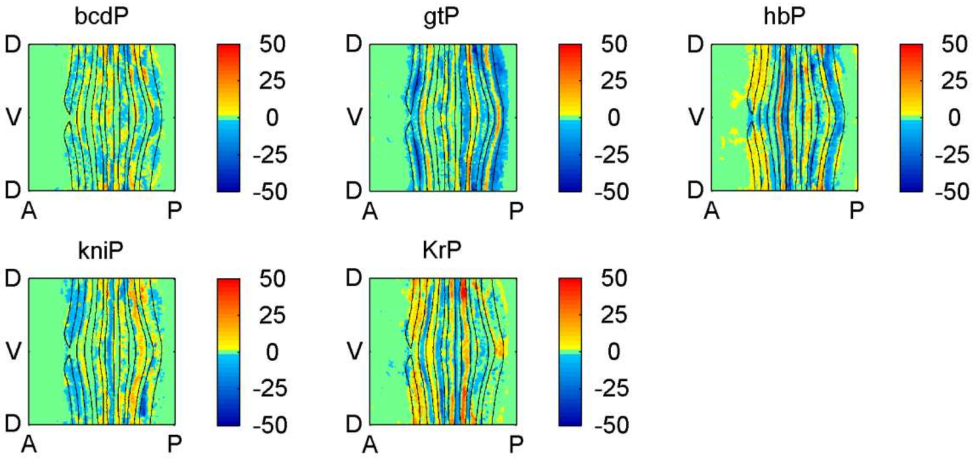

Factor Activity of NODE Model with Fixed Window Sizes

Stage 5:0-3

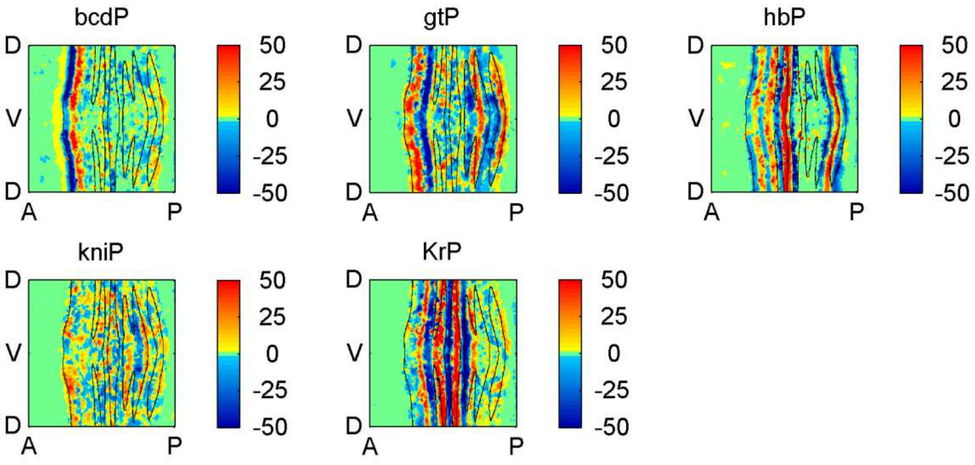

Stage 5:4-8

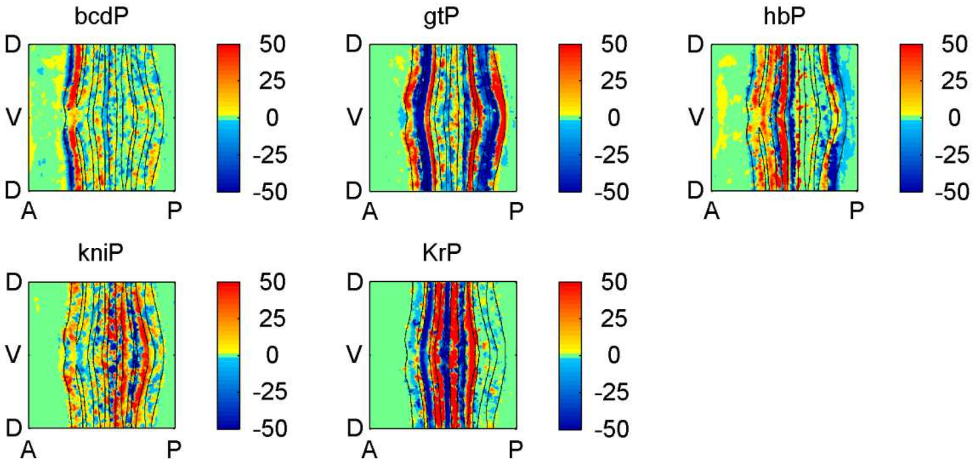

Stage 5:9-25

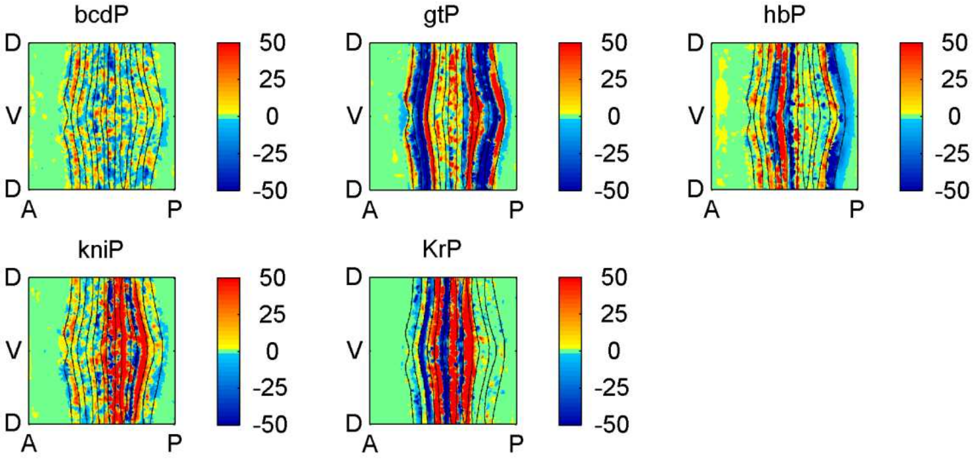

Stage 5:26-50

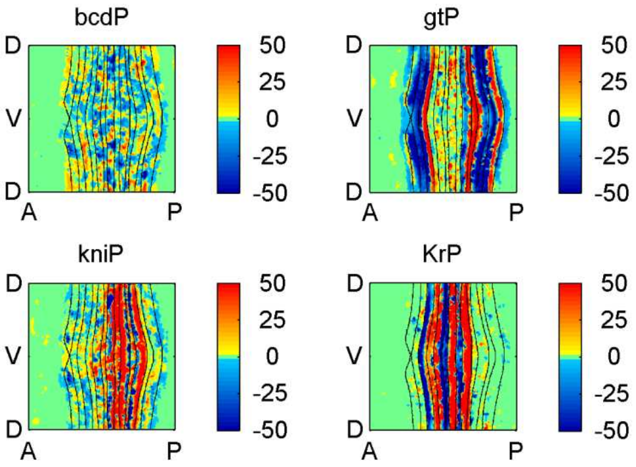

Stage 5:51-75

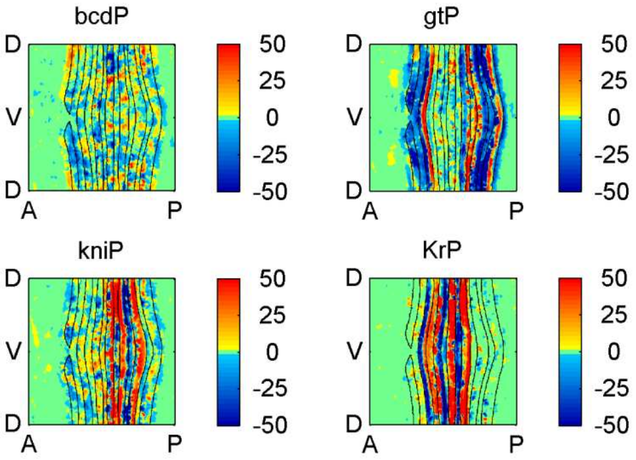

Factor Activity of Spatial-Correlation Model with Auto-Selected Window Sizes

Stage 5:0-3

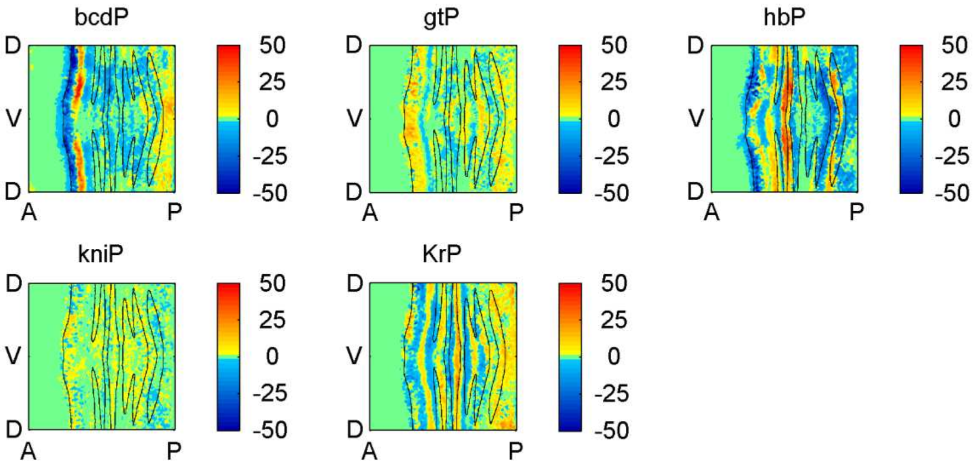

Stage 5:4-8

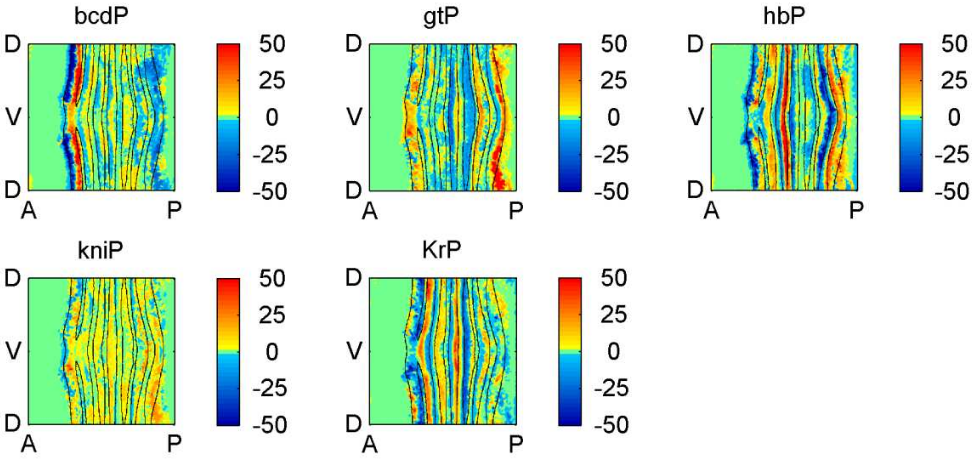

Stage 5:9-25

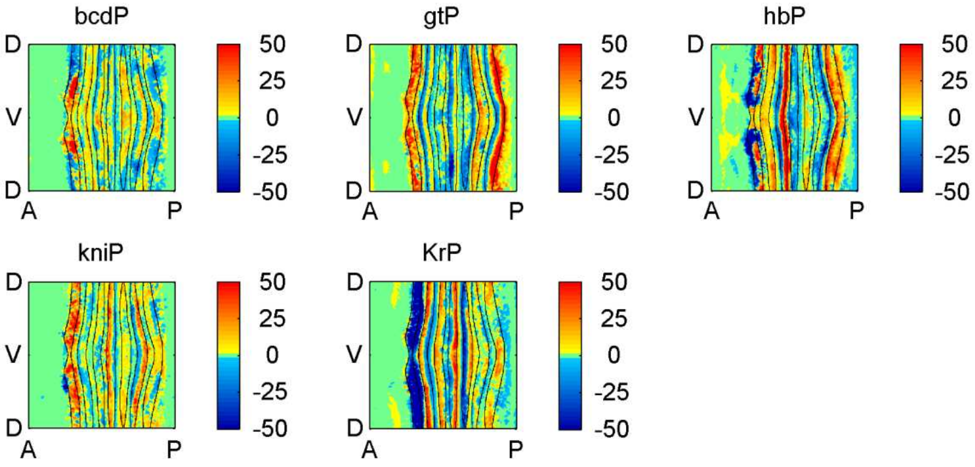

Stage 5:26-50

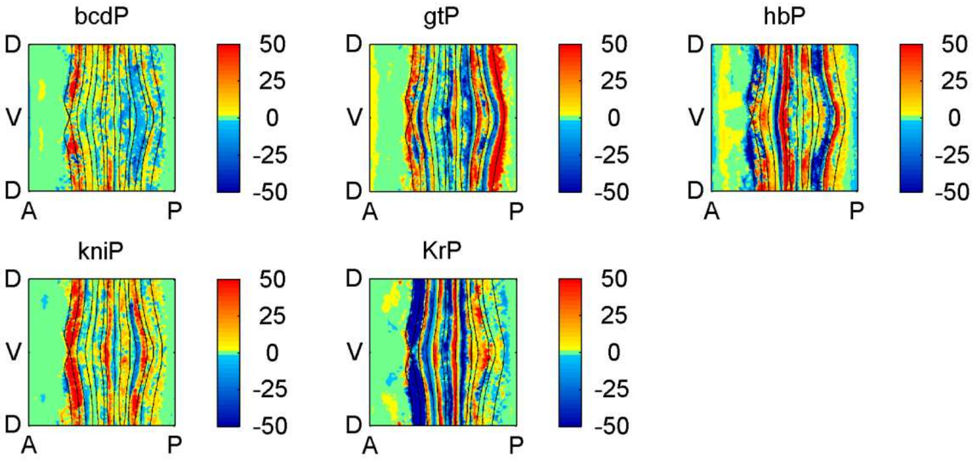

Stage 5:51-75

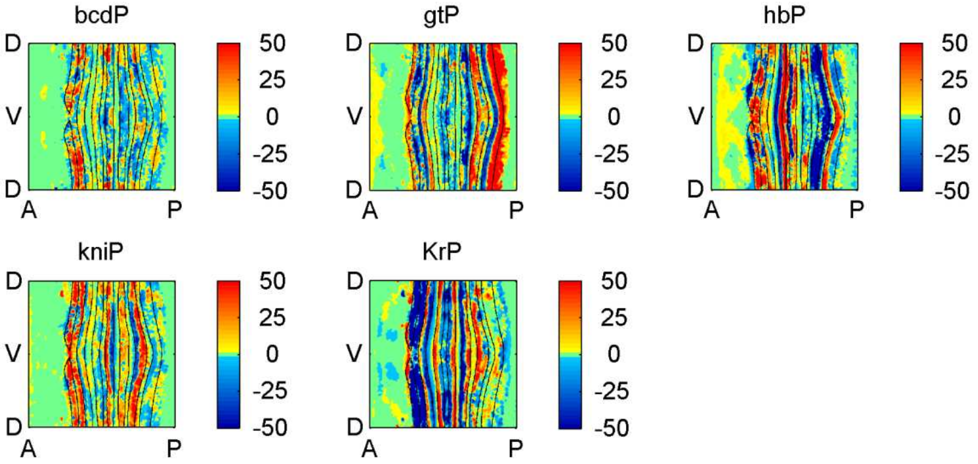

Factor Activity of Spatial-Correlation Model with Fixed Window Sizes

Stage 5:0-3

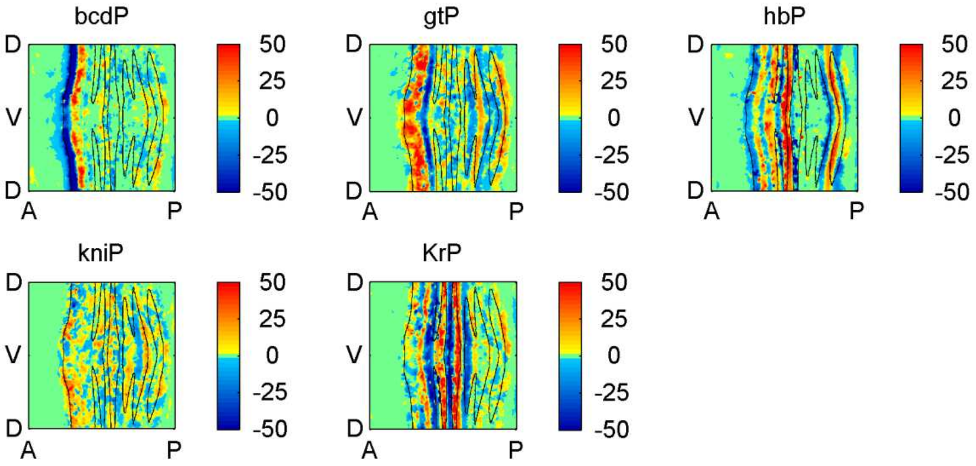

Stage 5:4-8

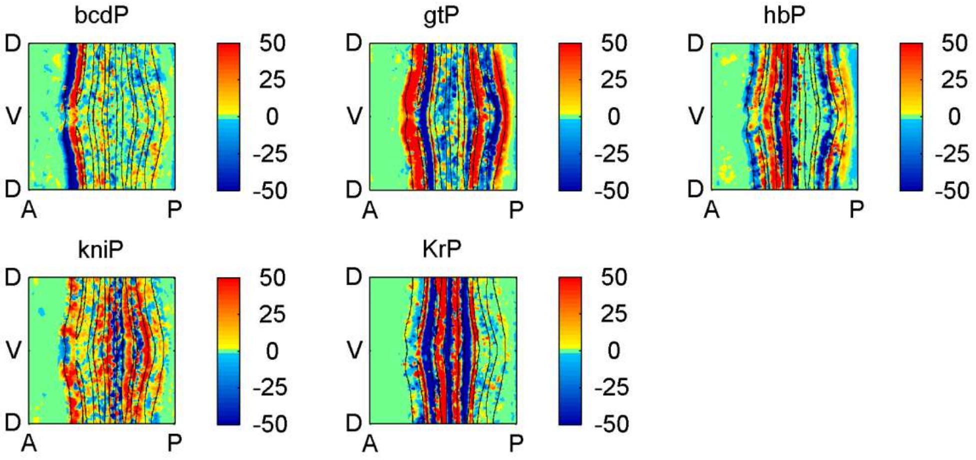

Stage 5:9-25

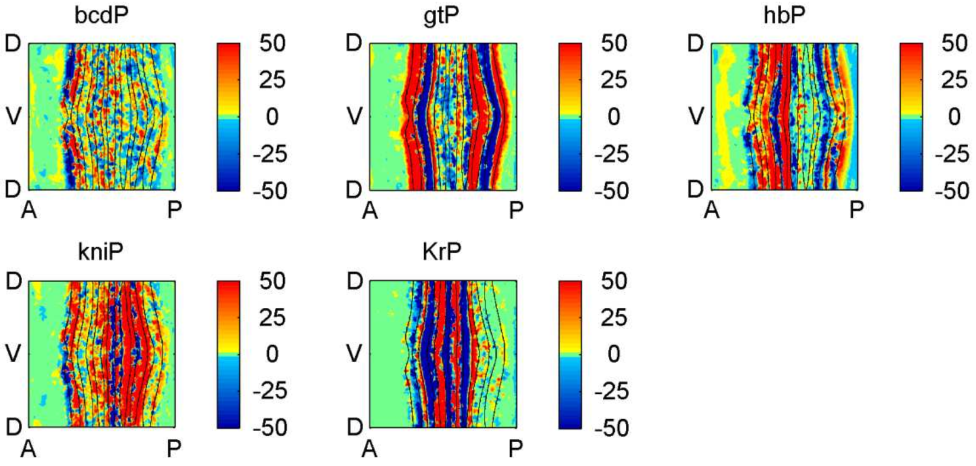

Stage 5:26-50

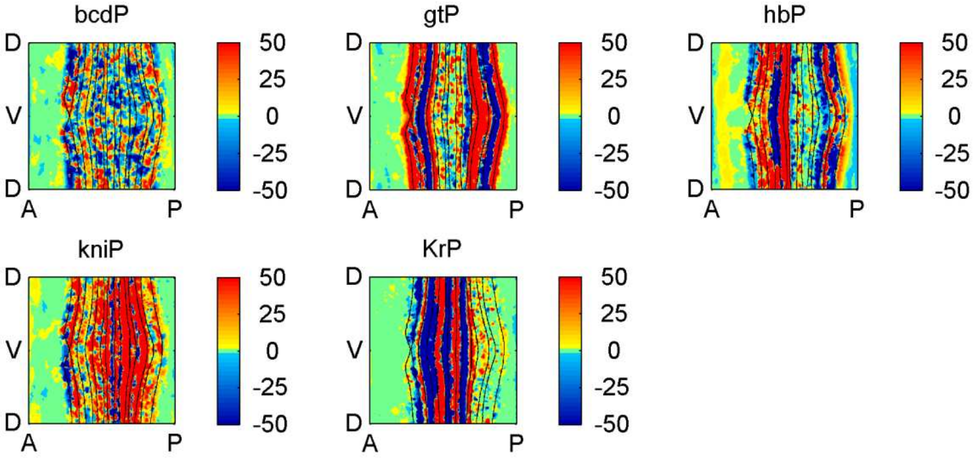

Stage 5:51-75

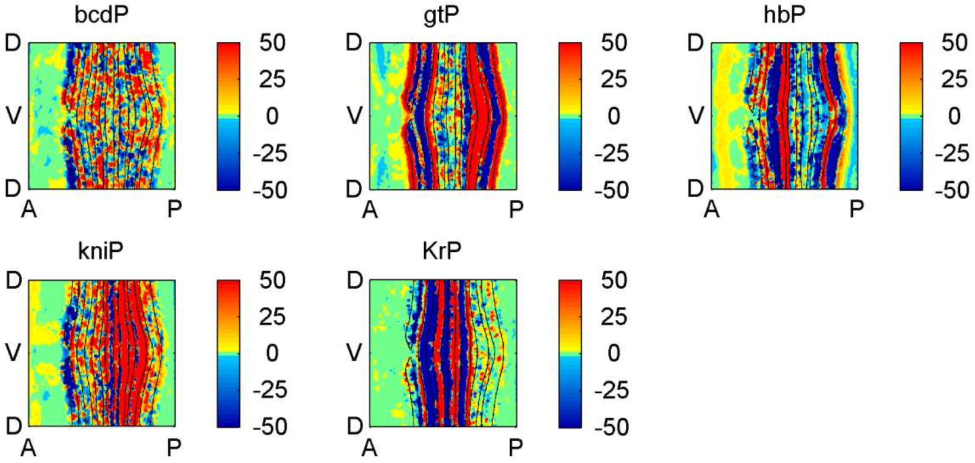

Supplement: Additional file 1 — Supplementary material. Full set of Factor Activity plots generated with both cross-validation-selected and fixed window sizes. [file 1471-2105-11-413-S1.PDF]
